# MuSK-antibodies are associated with worse outcome in myasthenic crisis requiring mechanical ventilation

**DOI:** 10.1007/s00415-021-10603-9

**Published:** 2021-05-10

**Authors:** Nicole König, Henning R. Stetefeld, Christian Dohmen, Philipp Mergenthaler, Siegfried Kohler, Silvia Schönenberger, Julian Bösel, De-Hyung Lee, Stefan T. Gerner, Hagen B. Huttner, Hauke Schneider, Heinz Reichmann, Hannah Fuhrer, Benjamin Berger, Jan Zinke, Anke Alberty, Ingo Kleiter, Christiane Schneider-Gold, Christian Roth, Juliane Dunkel, Andreas Steinbrecher, Andrea Thieme, Felix Schlachetzki, Ralf A. Linker, Klemens Angstwurm, Andreas Meisel, Bernhard Neumann, for The German Myasthenic Crisis Study Group, Kornelius Fuchs, Berthold Schalke, Amelie Vidal, Izabela Brachaczek, Jana Maidhof, Arno Wenke, Manuel Hagen, Jan Rahmig, Eik Schimmel, Wolf Niesen, Christine Fahrendorf

**Affiliations:** 1 Department of Neurology, University of Regensburg, Bezirksklinikum Regensburg, Universitaetsstraße 84, 93051 Regensburg, Germany; 2 Department of Neurology, University of Cologne, Cologne, Germany; 3 Department of Neurology, LVR-Klinik Bonn, Bonn, Germany; 4 NeuroCure Clinical Research Center, Charité, Universitätsmedizin Berlin, Berlin, Germany; 5 Departments of Neurology and Experimental Neurology, Center for Stroke Research Berlin, Charité, Universitätsmedizin Berlin, Berlin, Germany; 6 Berlin Institute of Health (BIH), Berlin, Germany; 7 Department of Neurology, Heidelberg University Hospital, Heidelberg, Germany; 8 Department of Neurology, Klinikum Kassel, Kassel, Germany; 9 Department of Neurology, University Hospital Erlangen, Erlangen, Germany; 10 Department of Neurology, University Hospital, Technische Universität Dresden, Dresden, Germany; 11 Department of Neurology, University Hospital Augsburg, Augsburg, Germany; 12 Clinic of Neurology and Neurophysiology, Medical Center, Faculty of Medicine, University of Freiburg, Freiburg, Germany; 13 Hans Berger Department of Neurology, Jena University Hospital, Jena, Germany; 14 Department of Neurology, Kliniken Maria Hilf GmbH Moenchengladbach, Mönchengladbach, Germany; 15 St. Josef-Hospital, Department of Neurology, Ruhr-University Bochum, Bochum, Germany; 16 Marianne-Strauß-Klinik, Behandlungszentrum Kempfenhausen Für Multiple Sklerose Kranke gGmbH, Berg, Germany; 17 Department of Neurology, DRK-Kliniken Nordhessen, Kassel, Germany; 18 Department of Neurology, Phillips University of Marburg, Marburg, Germany; 19 Department of Neurology, Helios Klinikum Erfurt, Erfurt, Germany

**Keywords:** Myasthenia gravis, Myasthenic crisis, Autoimmune diseases, Antibody status, MuSK-antibodies, Outcome

## Abstract

Myasthenic crisis (MC) is a life-threatening condition for patients with myasthenia gravis (MG). Muscle-specific kinase-antibodies (MuSK-ABs) are detected in ~ 6% of MG, but data on outcome of MuSK-MCs are still lacking. We made a subgroup analysis of patients who presented with MC with either acetylcholine-receptor-antibody positive MG (AchR-MG) or MuSK-MG between 2006 and 2015 in a retrospective German multicenter study. We identified 19 MuSK-AB associated MCs in 15 patients and 161 MCs in 144 patients with AchR-ABs only. In contrast to patients with AchR-AB, MuSK-AB patients were more often female (*p* = 0.05, OR = 2.74) and classified as Myasthenia Gravis Foundation of America-class IV before crisis (*p* = 0.04, OR = 3.25). MuSK-AB patients suffer more often from multiple chronic disease (*p* = 0.016, OR = 4.87) and were treated more invasively in terms of plasma exchanging therapies (not significant). The number of days of mechanical ventilation (MV) (43.0 ± 53.1 vs. 17.4 ± 18; *p* < 0.0001), days on an intensive care unit (ICU) (45.3 ± 49.5 vs. 21.2 ± 19.7; *p* < 0.0001), and hospital-length of stay (LOS) (55.9 ± 47.6 vs. 28.8 ± 20.9 days; *p* < 0.0001) were significantly increased in MuSK-MC. Remarkable is that these changes were mainly due to patients with MusK-ABs only, whereas patients’ outcome with both antibodies was similar to AchR-MCs. Furthermore, our data showed a shortened duration of MV after treatment with plasma exchanging therapies compared to treatment with intravenous immunoglobulin in MuSK-MCs. We conclude that MuSK-AB-status is associated with a longer need of MV, ICU-LOS, and hospital-LOS in MC, and therefore recommend early initiation of a disease-specific therapy.

## Introduction

MG is an autoimmune disorder with antibodies targeting the postsynaptic endplate causing muscle weakness. Whereas ~ 85% of patients are tested positive for AchR-ABs, MuSK-ABs are detected in around ~ 6% of all patients and in around 36–37% of AchR-AB-negative patients, representing the second largest cohort in MG [1, 2]. In MuSK-AB positive MG, previous studies have shown characteristic differences compared to AchR-MG: In brief, MuSK-MG predominantly appears in women, who show weakness in mostly cranial and bulbar muscles, commonly with an acute onset and a tendency to rapid progression in comparison to AchR-MG [2, 3]. Furthermore, recent studies indicated MuSK-MG as the more severe form as up to a half develop a myasthenic crisis (MC) in their disease course [4]. Moreover, a worse long-term outcome accompanied by relevant deficits might be suggestive in MuSK-MG compared to AchR-MG [5]. Whereas the use of cholinesterase inhibitors, maintaining a high potassium serum level, performing thymectomy, and adjusting immune modulating drugs, such as steroids, azathioprine, or mycophenolate mofetil, have been entrenched therapeutic strategies for AchR-MG, especially rituximab-infusions were lately shown to be highly effective in the remission of symptomatic MuSK-MG patients and maintaining a more stable disease course. Furthermore, it enables the reduction or withdrawal of other immunosuppressive medications, foremost the use of steroids [5, 6]. As rituximab is introduced sometime in the later disease course, MuSK-MG patients might still be on a higher risk of developing MC.

MC is a potential reversible but life-threatening condition, mostly provoked by infections, inadequate treatment, or following surgery. It appears in around 15–20% of MG patients in the first 2 years after diagnosis [7, 8]. Characteristic symptoms are extensive weakness, dyspnea, and dysphagia, which might result in respiratory insufficiency. Concerning the management of MC, early intubation to secure airways, as well as the combination of symptomatic treatment with intravenous choline esterase inhibitors and an acute causal treatment (Plasma exchange/Immunoadsorption/Intravenous Immunoglobulins) have led to a decline of mortality from around 40% until the early 1960s to a usually range in between 5 and 12% in recent studies [8–11]. Plasma exchange, Immunoadsorption, and Intravenous Immunoglobulins have shown to be comparable in treatment efficacy enabling a similar duration of treatment effect [12]. With respect to this current consensus, further studies have discussed a more beneficial effect of plasma exchanging therapies in MuSK-MG [2, 13]. In addition to that, Lazaridis et al. [14] detected a significant reduction of MuSK-AB serum levels using the more specific Immunoadsorption in an in vivo animal model.

While most of the previous studies focused on characteristics, therapeutic management, and outcome of MC, there are less data about the influence of patients’ antibody status on the development and course of MC. Therefore, this study was performed to assess differences of clinical features, therapeutic management, and outcome between AChR-MC and MuSK-MC.

## Methods

### Study design and patient selection

Subgroup analysis of MuSK-MC needing mechanical ventilation who were compared to AchR-MC treated at 12 German Departments of Neurology with specialized Neuro-Intensive Care Units (NICU) or neurologically associated interdisciplinary ICU. All consecutive patients were analyzed retrospectively if they had MC and required mechanical ventilation. For identification, all patients discharged with the diagnosis of MG according to the International Classification of Diseases (ICD10: G70.0-70.3) who were treated and ventilated on an ICU between 2006 and 2015 were reviewed. MC was defined as an exacerbation of myasthenic symptoms with bulbar and/or general weakness requiring mechanical ventilation. Diagnosis of MG had to be established according to national guidelines [15] and confirmed by specific tests (antibody testing or repetitive stimulation or improvement after cholinergic medication). Patients with cholinergic crisis, Lambert–Eaton syndrome, and myasthenic syndromes other than MG (such as congenital MG) were excluded as well as those who required mechanical ventilation due to other reasons than MG (e.g., heart failure or after surgery) and if mechanical ventilation was initiated within 4 weeks after thymectomy to exclude patients with postthymectomy crisis. Episodes of MC were counted separately if patients were discharged in their prehospital status and if new triggers for the next crisis could be determined.

For this subgroup analysis, matching of three AchR-MC to one MuSK-MC was done in following priority: sex, age, onset-type, Myasthenia Gravis Foundation of America (MGFA)-Class before crisis and where possible by complications of MC. If an exact matching was not possible, disadvantageous matching for AchR-MC was done (especially for age). For the analysis and matching, we only included AchR-patients treated at the same centers than MuSK-MCs, since MuSK-MCs were mainly treated at large MG centers with more experience in the treatment of MC.

### Data acquisition

Data on baseline demographics, clinical information, medication, and comorbidities were obtained through medical charts and institutional databases. Characteristics reviewed included antibody status, evidence of thymoma, and Myasthenia Gravis Foundation of America (MGFA)- Score prior to MC. Assessed treatment regimens were intravenous immunoglobulins (IVIG), Plasma exchanging therapy (PE), Immunoadsorption (IA), use of intravenous pyridostigmine, and continuous potassium infusion. Analyzed data regarding the clinical course of the crisis included time at ICU, days in hospital, duration of mechanical ventilation, in-hospital mortality, and referral/discharge.

### Statistics

GraphPad Prism 5^®^ (GraphPad Software, La Jolla, USA) was used for statistical analysis. Data were presented as mean (standard deviation and sometimes range) or total number with percentage. Group-comparison was tested with either Student’s *t* test, Fisher’s exact test [with odds ratios (OR)], or one-way ANOVA (with Newman-Keuls Multiple Comparison Test), respectively. The significance level was set to $\alpha =0.05$ both-sided.

## Results

### Characteristics of study group

The patient sample consisted of 19 independent MuSK-MCs in 15 patients (eight patients also had AChR-ABs, 7 solely MuSK-ABs) and 161 MCs in 144 patients with solely AChR-ABs needing MV (Table 1). Patients with MuSK-ABs were significantly more likely to be female (63.2% vs. 38.5%; *p* = 0.05; OR = 2.74), had multiple comorbidities (26.3% vs. 6.8%; *p* = 0.016; OR = 4.87), and had more often MGFA-Class IV before crisis (31.6% vs. 12.4%; *p* = 0.04; OR = 3.25). Furthermore, MuSK-MCs were treated more invasively, i.e., with Plasma exchange (PE) or Immunoadsorption (IA) (68.4% vs. 44.7%; *p* = 0.056; OR = 2.68) or with the combination of intravenous immunoglobulin (IVIG) and PE or IA (26.3% vs. 15.6%; *p* = 0.32; OR = 1.94) compared to AChR-MCs. An important result was that days of MV (43.0 ± 53.1 vs. 17.4 ± 18; *p* < 0.0001), ICU-LOS (45.3 ± 49.5 vs. 21.2 ± 19.7; *p* < 0.0001), and hospital-LOS (55.9 ± 47.6 vs. 28.8 ± 20.9; *p* < 0.0001) were significantly higher in MuSK-MCs. First-line therapy with PE/IA tends to shorten the duration of MV compared to treatment with IVIGs in MuSK-MCs (30.2 ± 29.8 vs. 51.3 ± 65.5; *p* = 0.36), although the former were older (69.6 vs. 59.4 years; *p* = 0.25).

**Table 1:** Comparison of episodes of myasthenic crisis with MuSK- and AChR-ABs

Myasthenic crisis	ACh-Rec.-positive (*n* = 161)	MuSK—positive (*n* = 19)	*p* value	Odds ratio
Age	66.8 ± 15.6 (14 – 88)	66.0 ± 17.7 (28 – 82)	0.83	
Age ≤ 50 years	22 (13.7%)	4 (21.1%)	0.49	1.69
**Male/female**	**99/62**	**7/12**	**0.05**	**2.74**
**Pulmonary disease**	**35 (21.7%)**	**9 (47.4%)**	**0.02**	**3.24**
Heart disease	66 (41%)	8 (42.1%)	1.00	1.05
Diabetes mellitus	48 (29.8%)	2 (10.5%)	0.10	0.28
Tumour (other than Thymoma)	23 (14.3%)	6 (31.6%)	0.09	2.78
Dialysis	2 (1.2%)	0 (0%)	1.00	1.64
Smoker	12 (7.5%)	1 (5.3%)	1.00	0.69
Alcohol addicted	5 (3.1%)	0 (0%)	1.00	0.73
**≥ 3 diseases (Kidney, Heart, Lung, Diabetes, Tumour)**	**11 (6.8%)**	**5 (26.3%)**	**0.016**	**4.87**
Myasthenia gravis				
Early onset	22 (13.9%; 3 unknown)	4 (22.2%; 1 unknown)	0.31	1.75
Late onset	136 (86.1%)	14 (77.8%)	0.31	0.57
Paraneoplastic MG (Thymoma)	58 (36%)	4 (21.1%)	0.31	0.47
Thymus hyperplasia	5 (3.1%)	0	1.00	0.73
MGFA-classification before crisis				
First manifestation of MG	35 (21.7%)	3 (15.8%)	0.77	0.68
Class I	10 (6.2%)	0 (0%)	0.60	0.37
Class II	42 (26.1%)	4 (21.1%)	0.78	0.76
Class III	40 (24.8%)	5 (26.3%)	1.00	1.08
** Class IV**	**20 (12.4%)**	**6 (31.6%)**	**0.04**	**3.25**
Unknown	14 (8.7%)	1 (5.3%)		
Status before crisis				
Independent at home	71 (44.1%)	6 (31.6%)	0.34	0.58
At home dependent on help	19 (11.8%)	3 (15.8%)	0.71	1.41
In a care facility or hospital	50 (31.1%)	9 (47.4%)	0.20	2.00
Unknown	21 (13.0%)	1 (5.3%)		
Cause of crisis				
Infection	85 (52.8%)	10 (52.6%)		
First episode	34 (21.1%)	3 (15.8%)		
Poor treatment compliance	9 (5.6%)	1 (5.3%)	n.s	
Intake of contraindicated medication	2 (1.2%)	0 (0%)		
Idiopathic/unknown	33 (20.5%)	5 (26.3%)		
Therapy				
IVIG	92 (57.5%; 1 unknown)	9 (47.4%)	0.47	0.68
Plasma exchange/immunoadsorption	72 (44.7%)	13 (68.4%)	0.056	2.68
PE or IA as first-line therapy	49 (30.4%)	10 (52.6%)	0.07	2.56
IVIG + plasma exchange or Immunoadsorption	25 (15.6%)	5 (26.3%)	0.32	1.94
Continuous pyridostigmine infusion	63 (39.1%)	7 (36.8%)	1.00	0.91
Continuous potassium infusion	66 (41%)	6 (31.6%)	0.47	0.66
Complications				
CPR	16 (9.9%)	2 (10.5%)	1.00	1.06
Pneumonia	86 (53.4%)	13 (68.4%)	0.23	1.89
Sepsis	27 (16.8%)	6 (31.6%)	0.12	2.29
Outcome				
** Days of mechanical ventilation at ICU**	**17.4 ± 18 (1–119)**	**43.0 ± 53.1 (4–219)**	**< 0.0001**	
** Days at ICU**	**21.2 ± 19.7 (1–135)**	**45.3 ± 49.5 (6–219)**	**< 0.0001**	
** Days in hospital**	**28.8 ± 20.9 (2–144)**	**55.9 ± 47.6 (11–219)**	**< 0.0001**	
In-hospital mortality	16 (9.9%)	3 (15.8%)	0.43	1.70

Age, “Days of mechanical ventilation at ICU”, “Days at ICU” and “Days in hospital” are depicted as mean ± Standard Deviation and range, other parameters are total number with percentage in brackets. *MGFA* Myasthenia Gravis Foundation of America, *MG* Myasthenia Gravis, *IVIG* intravenous immunoglobulin, *PE* plasma exchange, *IA* immunoadsorption, *CPR* Cardio Pulmonal Resuscitation, *n.s.* not significant. *T* test was used for statistic analysis of age-differences and for comparison of “Days of mechanical ventilation at ICU”, “Days at ICU” and “Days in hospital”. For other parameters, Fisher’s exact test with odds ratio was used

Significant result (*p* ≤ 0.05) are shown in bold letters

### MuSK-ABs are associated with prolonged MV and ICU-LOS in matched analysis

To exclude confounding variables, we matched one MusK-positive crisis to three AChR-positive crises for most known risk factors for prolonged mechanical ventilation (7). The groups did not differ in age, sex, number of multiple chronic comorbidities, percentage of late-onset MG, MGFA-classification before crisis, and complications of MC (Table 2). More patients with MuSK-MC were in nursing care facilities or hospitals before crisis (47.4% vs. 28.1%; *p* = 0.16; OR = 2.31). MuSK patients were treated more frequently with PE or IA compared to the matched AChR-AB group (68.4% vs. 42.1%; *p* = 0.06; OR = 2.98). We found no significant differences in co-treatment with prednisolone (57% vs. 52%; *p* = 0.79; OR = 1.23) or in the frequency of treatment with cortisone-sparing strategies (azathioprine, rituximab, MTX, or mycophenolate mofetil) (42% vs. 40%; *p* = 1.0; OR = 1.08) at the timepoint of the MC. The use of Rituximab was significantly higher in patients with MuSK-ABs (15.8% vs. 1.8%; *p* = 0.04; OR = 10.9). In 28.1% and 31.6%, respectively, an additional treatment was not done or unknown in our cohort.

**Table 2:** Comparison of matched MuSK- and AChR-AB positive myasthenic crisis requiring reintubation

Myasthenic crisis	ACh-Rec.-positive (*n* = 57)	MuSK—positive (*n* = 19)	*p* value	Odds ratio
Age	66.3 ± 17.0 (24–88)	66.0 ± 17.7 (28–82)	0.95	
Male/female	22/35	7/12	1.00	1.08
≥ 3 diseases (Kidney, Heart, Lung, Diabetes, Tumour)	14 (24.6%)	5 (26.3%)	1.00	1.10
Late-onset Myasthenia gravis	44 (77.2%)	14 (77.8%; 1 unknown)	1.00	1.03
MGFA-classification before crisis				
First manifestation of MG	9 (15.8%)	3 (15.8%)	1.00	1.00
Class I	0 (0%)	0 (0%)		
Class II	14 (24.6%)	4 (21.1%)	1.00	0.82
Class III	16 (28.1%)	5 (26.3%)	1.00	0.92
Class IV	16 (28.1%)	6 (31.6%)	0.78	1.18
Unknown	2 (3.5%)	1 (5.3%)	1.00	1.54
Status before crisis				
Independent at home	25 (43.9%)	6 (31.6%)	0.42	0.59
At home dependent on help	5 (8.8%)	3 (15.8%)	0.40	1.96
In a care facility or hospital	16 (28.1%)	9 (47.4%)	0.16	2.31
Unknown	11 (19.3%)	1 (5.3%)	0.27	0.23
Therapy				
IVIG	36 (63.2%)	9 (47.4%)	0.28	0.53
Plasma exchange/Immunoadsorption	24 (42.1%)	13 (68.4%)	0.06	2.98
PE or IA as first-line therapy	18 (31.6%)	10 (52.6%)	0.11	2.38
IVIG + plasma exchange or Immunoadsorption	9 (15.8%)	5 (26.3%)	0.32	1.89
Continuous pyridostigmine infusion	24 (42.1%)	7 (36.8%)	0.79	0.80
Continuous potassium infusion	23 (40.4%)	6 (31.6%)	0.59	0.68
Complications				
CPR	9 (15.8%)	2 (10.5%)	0.72	0.63
Pneumonia	34 (59.7%)	13 (68.4%)	0.59	1.47
Sepsis	14 (24.6%)	6 (31.6%)	0.56	1.41
Outcome				
** Days of mechanical ventilation at ICU**	**18.8 ± 21.9 (1–119)**	**43.0 ± 53.1 (4–219)**	**0.0078**	
** Days at ICU**	**22.3 ± 21.0 (1–119)**	**45.3 ± 49.5 (6–219)**	**0.0067**	
** Days in hospital**	**26.9 ± 20.6 (2–119)**	**55.9 ± 47.6 (11–219)**	**0.0006**	
In-hospital mortality	5 (8.8%)	3 (15.8%)	0.40	1.95

Age is depicted as mean ± Standard Deviation and range, other parameters are total number with percentage in brackets. *MGFA* Myasthenia Gravis Foundation of America, *MG* Myasthenia Gravis, *IVIG* intravenous immunoglobulin, *PE* plasma exchange, *IA* immunoadsorption, *CPR* Cardio Pulmonal Resuscitation. *T* test was used for statistic analysis of age-differences. For other parameters, Fisher’s exact test with odds ratio was used

Significant result (*p* ≤ 0.05) are shown in bold letters

**Fig. 1:**
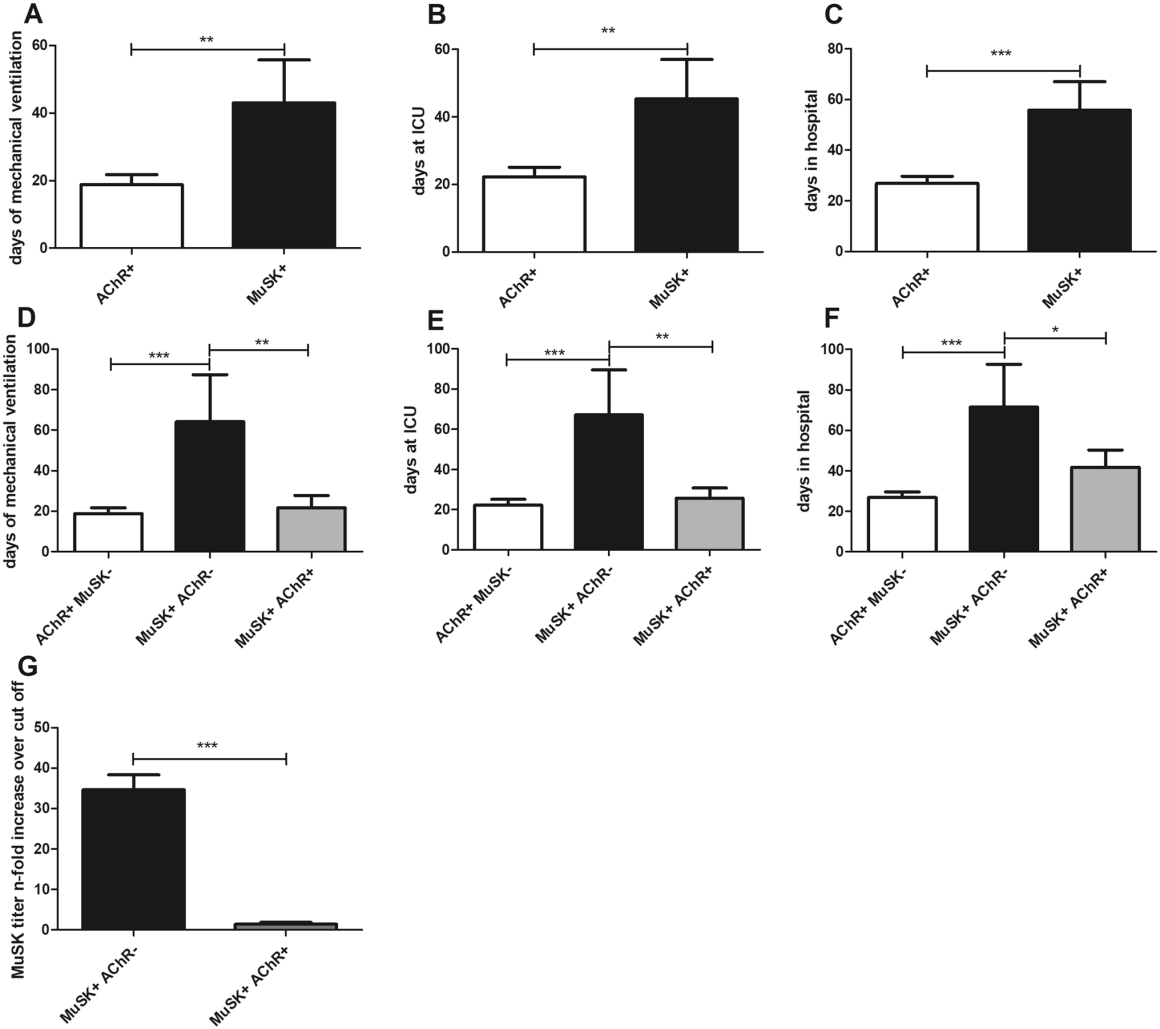
**a** Days of mechanical ventilation. **b** Days at ICU. **c** Days in hospital in 57 MCs with AChR-ABs matched to 19 MCs with MuSK-ABs. Bars show mean ± SD (*t *test). **d** Days of mechanical ventilation. **e** Days at ICU. **f** Days in hospital in 57 MCs with AChR-ABs, 10 MCs with both MuSK- and AChR-Abs, and 9 MCs only having MuSK-ABs. Bars show mean ± SD (ANOVA). **g** n-fold increase of MuSK-titers over cut off of five patients with both MuSK- and AChR-ABs and five patients only having MuSK-ABs. Bars show mean ± SD (*t* test). *p < 0.05, **p < 0.01, ***p < 0.001

Furthermore, days of MV (43.0 ± 53.1 vs. 18.8 ± 21.9; *p* = 0.0078), ICU-LOS (45.3 ± 49.5 vs. 22.3 ± 21.0; *p* = 0.0067), and hospital-LOS (55.9 ± 47.6 vs. 26.9 ± 20.6; *p* = 0.0006) were significantly higher in MuSK-MCs compared to patients with AChR-ABs (Fig. 1a–c). After discharge, patients with MuSK-ABs needed MV to a similar degree (31.3% vs. 21.2%; *p* = 0.50; OR = 1.69) and did not show a higher mortality (15.8% vs. 8.8%; *p* = 0.40; OR = 1.95).

Interestingly, this difference was mainly due to the 9 MCs of patients with MuSK-AB having no additional AChR-ABs, who needed more days of MV (64.2 ± 65.6 vs. 21.8 ± 16.9), ICU-LOS (67.0 ± 63.4 vs. 25.7 ± 15.3) and hospital-LOS (71.4 ± 60.0 vs. 41.9 ± 25.5) compared to patients who had both MuSK- and AChR-ABs (Fig. 1d–f). Furthermore, MuSK-titers during MCs were higher in patients with solely MuSK-ABs (Fig. 1g). Of notice, patients with MuSK-ABs and without AChR-ABs were older (67.8 vs. 64.3 years) than patients with both MusK- and AChR-Abs. Moreover, patients with MuSK-ABs had a high risk to die (15.8% vs. 8.8%; *p* = 0.40; OR = 1.95) and surviving patients were more often discharged still needing MV (31.3% vs. 21.2%; *p* = 0.50; OR = 1.69).

## Discussion

In our large cohort of MC needing MV, we observed that MuSK-positive antibody status was associated with prolonged MV, ICU-LOS, and hospital-LOS reflecting a more severe course of MC.

Since the detection of MuSK-ABs in MG, only few studies on the outcome of MC needing MV have been published yet [1]. These studies only included small numbers of patients with MuSK-ABs or did not specify the antibody status during MC. Until now, data on the effect of antibody status on the outcome of severe MC are lacking. Other studies with a broader study population classified MuSK-MG as more severe form of MG with lower occurrence of clinical stable remission [4, 16], which is in concordance with our findings of a higher proportion of MGFA-class IV before crisis and more unfavorable outcome during MC, including a higher mortality.

Focusing on the therapeutic management, a similar number of patients in both groups received intravenous pyridostigmine. MuSK-ABs belong to IgG4-subclass, which may reduce the density of acetylcholine receptors as well as postsynaptic acetylcholine sensitivity [17]. Consequently, the effect of pyridostigmine in patients with MuSK-MG can be questioned. Other clinical studies reported a non-responsiveness of pyridostigmine in MuSK-MG of up to 71% [16, 18]. Thus, outcome in MC might also be unfavorably influenced by a reduced effect of acetylcholinesterase inhibitors specifically in MuSK-MG.

In MC, the inclusion of immunomodulatory therapies is unavoidable. Comparing our subgroups, physicians more frequently decided for a therapy with PE/IA in MuSK-MCs, whereas a higher number of AChR-MCs received IVIGs. This might be explained by individual treatment pathways in our participating centers, availability of treatment options, and due to its preferred listing in the German MG guidelines. Another explanation is that MuSK-AB status was known in 15 of 19 crises before treatment initiation, which could have led to a precautious treatment escalation in these patients. Our results showed that an early use of PE/IA reduced the days of MV in MuSK-MCs compared to IVIG-treatment. While plasma exchange has historically been the favorable treatment in MC, the latest consensus implies an equal effect of PE/IA vs. IVIGs [12]. Considering the low number of patients with MuSK-MG in those studies, separate studies on therapeutic effects of PE/IA vs. IVIGs on specifically MuSK-MC are obligatory to obtain individualized treatment approaches. In contrast to that, some studies already demonstrated a better symptomatic improvement in MuSK-MG after plasma exchanging therapies compared to IVIG, e.g., measured by the MGFA-classification, but data in MC are still lacking [2, 16]. Lately more and more studies discuss a potential favorable role of IA in MuSK-MC, as it is an antibody selective plasma exchange therapy with less risk for side effects compared to Plasmapheresis. This could be confirmed by first data from Lazaridis et al. [14, 19] indicating a significant and sufficient reduction of MuSK-AB serum level through IA. Barth et al. [12], who pointed out an equal effect of PE/IA vs. IVIG, stated that the presence of AChR-ABs predicted a better outcome compared to MuSK-MG or seronegative MG, which might furthermore empower a differentiation of decision-making in MC treatment due to its antibody status.

One of our most significant results was a longer need of mechanical ventilation (around 43 days) and therefore longer stay on ICU (around 45 days) in patients with MuSK-ABs compared to patients with AchR-ABs (around 17 days and 21 days), which also stresses its more aggressive disease course. Previous studies analyzing days of ventilation and treatment duration on ICU detected similar results on mostly AchR-MC (16.7–22.4 days), whereas a differentiation of antibody status is lacking due to low numbers of MuSK-MG patients in their cohort [10]. Around 25% of MG patients remained ventilated 1 month after mostly AchR-MC, which is similar than in our cohort [11]. Nevertheless, early diagnosis with pre-existing disease-specified treatment as well as treatment on a neurological ICU seem to be beneficial outcome measures [7, 10, 11].

What is interesting is that our findings were mainly caused by patients with solely MuSK-ABs, whereas patients with both antibody types had a similar outcome as solely AChR-AB MCs. This may be explained by the potentially higher effect of pyridostigmine in double-positive patients. Moreover, patients with MuSK-ABs were older, which is a potential confounder. Our data suggest that the titer of MuSK-ABs during MC plays a leading role here. Other studies have also shown a correlation of MuSK-AB-titer and MG severity in general [20]. Aguirre et al. [21] demonstrated similar results; they found a relation between AchR-AB titers and disease severity in the first 5 years of MG as well as they detected complement factor C5a significantly elevated in severe disease courses as a non-specific marker. Up to our knowledge, only a few studies, especially case reports**,** exist that repetitively measured antibody titers during disease course. Zouvelou and Psimenou [22] published a case of a double-positive young woman with especially high serum levels of MuSK-AB and a clinical course of a MuSK-MG and therefore development of severe MC. A few other cases have been reported with primary AchR-MG and symptomatic exacerbations with subsequent verification of MuSK-ABs. In our cohort, AB titers during current MC prior to treatment were only tested in 10 of 19 MCs and were detected significantly higher in solely MuSK-MCs. Possibly, double-positive patients have lower MuSK-AB-titers, but detailed studies on this patient cohort do not exist so far.

As we have focused on short-term outcome measures, at this point, we want to mention that long-term outcome seems beneficial regardless of antibody status. Especially in MuSK-MG, this might be highly influenced by the introduction of Rituximab in MG treatment and its sufficient reduction of MuSK-AB titers [5, 6].

Limitations of this study arise from its retrospective nature and of course of the small sample size, because MuSK-MC is rare. The high number of double-positive MCs was very surprising and could suggest that some of these patients were false anti-MuSK or false anti-AchR positives due to unspecificity of the test technique or positive results near the threshold range due to too sensitive tests. Nevertheless, our cohort represents by far the largest case series of MuSK-MC and provides interesting results.

We conclude that MuSK-AB-status is associated with a worse outcome in MC needing MV and we recommend early initiation of a focused therapy (especially PE/IA). Moreover, testing of MuSK-AB-titers during every MC may represent an important tool to estimate prolonged MV and the need for an intensified treatment.

## Data Availability

Anonymized data will be made available upon reasonable request.

## References

[1] Hoch W, McConville J, Helms S, Newsom-Davis J, Melms A, Vincent A (2001). Auto-antibodies to the receptor tyrosine kinase MuSK in patients with myasthenia gravis without acetylcholine receptor antibodies. Nat Med.

[2] Guptill JT, Sanders DB, Evoli A (2011). Anti-MuSK antibody myasthenia gravis: clinical findings and response to treatment in two large cohorts. Muscle Nerve.

[3] McConville J, Farrugia ME, Beeson D, Kishore U, Metcalfe R, Newsom-Davis J, Vincent A (2004). Detection and characterization of MuSK antibodies in seronegative myasthenia gravis. Ann Neurol.

[4] Baggi F, Andreetta F, Maggi L, Confalonieri P, Morandi L, Salerno F, Bernasconi P, Montomoli C, Barberis M, Mantegazza R, Antozzi C (2013). Complete stable remission and autoantibody specificity in myasthenia gravis. Neurology.

[5] Evoli A, Alboini PE, Damato V, Iorio R, Provenzano C, Bartoccioni E, Marino M (2018). Myasthenia gravis with antibodies to MuSK: an update. Ann N Y Acad Sci.

[6] Hehir MK, Hobson-Webb LD, Benatar M, Barnett C, Silvestri NJ, Howard JF, Howard D, Visser A, Crum BA, Nowak R, Beekman R, Kumar A, Ruzhansky K, Chen IA, Pulley MT, LaBoy SM, Fellman MA, Greene SM, Pasnoor M, Burns TM (2017). Rituximab as treatment for anti-MuSK myasthenia gravis: multicenter blinded prospective review. Neurology.

[7] Neumann B, Angstwurm K, Mergenthaler P, Kohler S, Schönenberger S, Bösel J, Neumann U, Vidal A, Huttner HB, Gerner ST, Thieme A, Steinbrecher A, Dunkel J, Roth C, Schneider H, Schimmel E, Fuhrer H, Fahrendorf C, Alberty A, Zinke J, German Myasthenic Crisis Study Group (2020). Myasthenic crisis demanding mechanical ventilation: a multicenter analysis of 250 cases. Neurology.

[8] Thomas CE, Mayer SA, Gungor Y, Swarup R, Webster EA, Chang I, Brannagan TH, Fink ME, Rowland LP (1997). Myasthenic crisis: clinical features, mortality, complications, and risk factors for prolonged intubation. Neurology.

[9] Alshekhlee A, Miles JD, Katirji B, Preston DC, Kaminski HJ (2009). Incidence and mortality rates of myasthenia gravis and myasthenic crisis in US hospitals. Neurology.

[10] Spillane J, Hirsch NP, Kullmann DM, Taylor C, Howard RS (2014). Myasthenia gravis–treatment of acute severe exacerbations in the intensive care unit results in a favourable long-term prognosis. Eur J Neurol.

[11] Ramos-Fransi A, Rojas-García R, Segovia S, Márquez-Infante C, Pardo J, Coll-Cantí J, Jericó I, Illa I, Myasthenia NMD-ES Study Group (2015). Myasthenia gravis: descriptive analysis of life-threatening events in a recent nationwide registry. Eur J Neurol.

[12] Barth D, Nabavi Nouri M, Ng E, Nwe P, Bril V (2011). Comparison of IVIg and PLEX in patients with myasthenia gravis. Neurology.

[13] Yamada C, Teener JW, Davenport RD, Cooling L (2015). Maintenance plasma exchange treatment for muscle specific kinase antibody positive myasthenia gravis patients. J Clin Apheresis.

[14] Lazaridis K, Baltatzidou V, Tektonidis N, Tzartos SJ (2020). Antigen-specific immunoadsorption of MuSK autoantibodies as a treatment of MuSK-induced experimental autoimmune myasthenia gravis. J Neuroimmunol.

[15] Wiendl H (2015) Diagnostik und therapie der myasthenia gravis und des Lambert-Eaton-Syndroms. Leitlinien für Diagnostik und Therapie in der Neurologie, 5

[16] Morren J, Li Y (2018). Myasthenia gravis with muscle-specific tyrosine kinase antibodies: a narrative review. Muscle Nerve.

[17] Klooster R, Plomp JJ, Huijbers MG, Niks EH, Straasheijm KR, Detmers FJ, Hermans PW, Sleijpen K, Verrips A, Losen M, Martinez-Martinez P, De Baets MH, van der Maarel SM, Verschuuren JJ (2012). Muscle-specific kinase myasthenia gravis IgG4 autoantibodies cause severe neuromuscular junction dysfunction in mice. Brain: J Neurol.

[18] Mehndiratta MM, Pandey S, Kuntzer T (2014). Acetylcholinesterase inhibitor treatment for myasthenia gravis. Cochrane Database Syst Reviews.

[19] Lazaridis K, Tzartos SJ (2020). Myasthenia gravis: autoantibody specificities and their role in MG management. Front Neurol.

[20] Bartoccioni E, Scuderi F, Minicuci GM, Marino M, Ciaraffa F, Evoli A (2006). Anti-MuSK antibodies: correlation with myasthenia gravis severity. Neurology.

[21] Aguirre F, Manin A, Fernandez VC, Justo ME, Leoni J, Paz ML, Villa AM (2020). C3, C5a and anti-acetylcholine receptor antibody as severity biomarkers in myasthenia gravis. Ther Adv Neurol Disord.

[22] Zouvelou V, Psimenou E (2020). AChR-and MuSK-positive myasthenia gravis: double trouble. J Neuroimmunol.

